# Impact of COVID‐19 on Subsequent Lung Function in Childhood: A Systematized Review

**DOI:** 10.1002/hsr2.72793

**Published:** 2026-07-07

**Authors:** Benedetta De Santis, Francesca Baldo, Giovanni Vento, Stefano Nobile

**Affiliations:** ^1^ Department of Mother, Child and Public Health, Division of Neonatology, Neonatal Unit Fondazione Policlinico Universitario “A. Gemelli” Rome Italy; ^2^ Department of Woman and Child Health and Public Health Università Cattolica del Sacro Cuore Rome Italy

**Keywords:** infant, pediatric, pulmonary function test, SARS‐CoV‐2

## Abstract

**Background and Aims:**

The SARS‐CoV‐2 infection can lead to transiently altered lung function in adults, but data is less clear in children. We aimed to summarize the available evidence about respiratory outcomes after COVID‐19 in childhood.

**Methods:**

We conducted a literature search on post‐COVID‐19 lung function tests (LFT) on the Medline and Cochrane databases, including studies published between 2019 and 2025.

**Results:**

Three hundred forty‐seven publications were identified, and 20 were selected. Results are presented in two sections, based on the duration of follow‐up. Most studies included spirometry, whereas many of them presented data from diffusing lung capacity for carbon monoxide, multiple breath nitrogen washout, fractional exhaled nitric oxide, impulse oscillometry, 6‐minute walking test, and interrupter resistance. Five papers described no association between COVID‐19 and respiratory outcomes, nor a link between the persistence of symptoms and the outcomes. Other authors reported a mild obstructive pattern which showed reversibility post bronchodilators, or a restrictive pattern, particularly when long‐COVID was evident. Some papers showed that pulmonary function may be influenced by the initial severity of the SARS‐CoV‐2 infection.

**Conclusion:**

Some studies reported no clear association between lung function impairment and previous history of COVID‐19. Other authors reported mild, transient consequences. A small number of papers concluded that severe infection, which is relatively rare in children, might affect pulmonary function. The main limitation of this review was the high heterogeneity of the included studies, especially regarding the COVID‐19 severity, the age groups, the LFT details, and the duration of follow‐up.

## 1 Introduction

Since the beginning of the coronavirus disease 19 (COVID‐19) pandemic, the severe acute respiratory syndrome coronavirus 2 (SARS‐CoV‐2) has infected over 777 million people and determined over 7 million deaths worldwide [1]. Both children and adults can be affected by SARS‐CoV‐2 infection although, compared to adults, children usually present with milder disease.

The severity of the infection ranges from asymptomatic to respiratory failure with a high mortality rate, particularly in older individuals or in those with co‐morbidities [2]. Compared to older individuals, a higher proportion of children present with asymptomatic or milder forms of disease [3]. Around 10%–15% of adults and children reported long‐COVID, whereas there are no data in infants [4]. Causes that have been hypothesized include endothelial damage, prothrombotic conditions, inflammation, immune dysregulation, microbiota dysbiosis, and dysfunctional neurological signaling. Regarding the respiratory consequences of COVID‐19, studies with lung function tests (LFT) or advanced imaging tools (functional computerized tomography or magnetic resonance imaging) reported that alterations in diffusion capacity and combinations of restrictive and obstructive patterns may be found in the first months after COVID‐19 in adults, with later improvement [5, 6].

Among the adult population, most studies found alterations of diffusion capacity and both restrictive and obstructive patterns, although these findings appear to be transient most of the time. In children, data is less clear: some authors reported a higher risk of altered lung function in children with a history of SARS‐CoV‐2 compared to controls. Nevertheless, these findings usually relate to children with severe symptoms during the acute infection. The aim of this review is to summarize the available evidence reporting on the respiratory outcomes after COVID‐19 in the pediatric population. A quality assessment of the included studies and a narrative synthesis of the evidence are presented.

## 2 Methods

We conducted a literature search on post‐COVID‐19 lung function tests in children on the Medline and Cochrane databases, including studies published between 2019 and 2025, using the following keywords: “COVID‐19”, “lung function test”, “children”, “SARS‐CoV‐2”, “pulmonary function test”, “pediatric”, “infant”. Their combinations were explored using the Boolean operator “AND” as appropriate. Inclusion criteria were: study population ≤ 18 years old with a previous diagnosis of SARS‐CoV‐2 infection, lung function as the primary outcome, and otherwise healthy children. The literature search was last updated on August 10, 2025. The inclusion criteria were English language and human species. Exclusion criteria were: non‐English language, studies conducted only in special populations (e.g., cystic fibrosis), animal species, case reports, case series, scientific letters, reviews, non‐systematic reviews, and editorials. As this was a systematized review, the review protocol was not registered. B.D.S. and S.N. independently conducted a literature search and appraisal. This study followed the relevant Preferred Reporting Items for Systematic

Reviews and Meta‐Analysis (PRISMA) standards. Since this study is based on a secondary literature review of published studies, it does not require additional ethical approval.

## 3 Results

**Figure 1:**
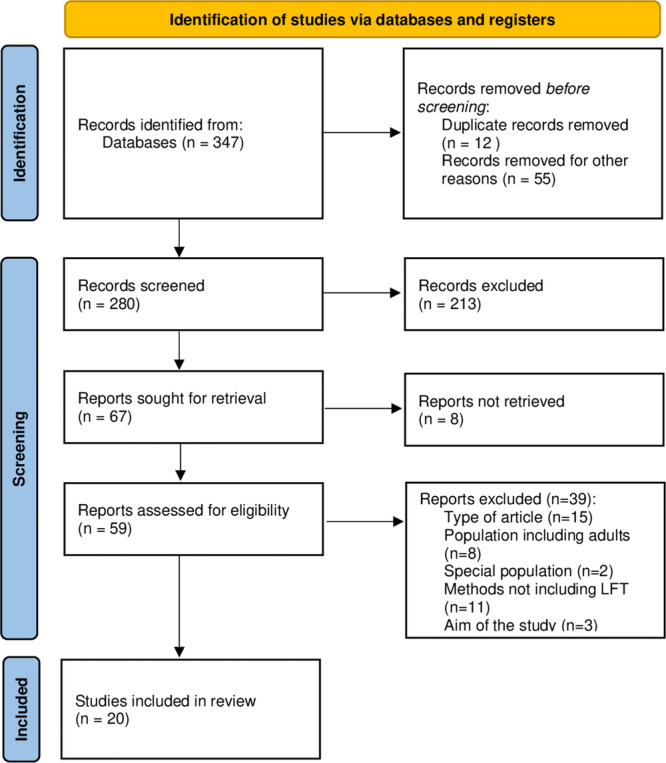
PRISMA flow diagram showing details about the search strategy and selection of studies.

A total of 347 publications were identified, and 20 were selected. Primary reasons for exclusion of the studies were: the type of study, the lack of inclusion of LFT as a primary outcome, and the included population (i.e., mixed populations of children and adults). An audit trail of the evidence is shown in Figure 1.

The results are presented in two sections, based on the duration of follow‐up. We arbitrarily considered 5 months' follow‐up as a cut‐off to define short versus long‐term follow‐up.

### 3.1 Studies With Short Term Follow Up

Ashkenazi et al. conducted a prospective cohort study on a population of 90 children under 18 years of age with a previous history of SARS‐CoV‐2 infection, performing pulmonary function tests in children who experienced persistent cardiorespiratory symptoms [7]. The median length of follow‐up was 112 days (range 33–410 days). The authors showed that 27 (45%) out of 60 children had a mild obstructive pattern (low Forced Expiratory Volume in 1 s–FEV1, air trapping), which exhibited reversibility after bronchodilators in more than half of the patients, suggesting that treatment with bronchodilators and inhaled corticosteroids might be effective in this clinical scenario.

Leftin Dobkin et al. focused on children aged 4–19 years (median age 13.1) with persistent respiratory symptoms following acute COVID‐19 [8]. Patients underwent spirometry, plethysmography, and 6‐minute walk test (6MWT). The authors described normal findings in spirometry and plethysmography, whereas the 6MWT, performed in only 9 children, revealed exercise intolerance and tachycardia in six patients (66%). Four out of fourteen patients had evidence of air trapping. The mean follow‐up was 3.2 ± 1.5 months after a SARS‐CoV‐2 positive polymerase chain reaction or confirmed close contact and suggestive clinical symptoms (range: 1.3–6.7 months). It should be noted that the paper described a convenience sample of children referred to the study center due to prolonged symptoms and that the prevalence of obesity and atopy was higher in the study population compared to the general population, limiting the generalizability of the results.

Bottino et al. focused on children (0.5–10.5 years old) with asymptomatic or mildly symptomatic SARS‐CoV‐2 infection [9]. This descriptive, prospective study on 16 patients assessed airway resistance by the interrupter technique test—Rint—(pre‐ and post‐bronchodilator assessment of airway resistance) in subjects aged 4–6 years, whereas forced spirometry and diffusing capacity of the lungs for carbon monoxide (DLCO) were performed in subjects aged ≥ 6 years. Duration of follow‐up was 49–91 days. None of the participants presented any abnormality in the lung function test results. As stated by the authors, this study only focuses on mild or asymptomatic infections, thus the present findings cannot be generalized to all children who recovered from a SARS‐CoV‐2 infection, especially those affected by the pediatric multisystem inflammatory syndrome. Furthermore, the sample size was small.

Knoke et al. performed various LFTs (multiple breath nitrogen washout test‐MBNW, body plethysmography, and diffusion capacity testing) on a population of 73 children aged 5 to 18 years old with serological or PCR‐based evidence of SARS‐CoV‐2 infection and on 45 SARS‐CoV‐2 seronegative controls [10]. This single‐center, cross‐sectional, prospective study did not report any significant difference in the frequency of abnormal pulmonary function patterns at follow‐up (average length of follow‐up: 2.6 months) between the two groups. Only two patients with persistent respiratory symptoms showed impaired pulmonary function, suggesting different pathophysiological mechanisms underlying the reported symptoms. Multivariate analysis revealed an association between severe SARS‐CoV‐2 infection and reduced forced vital capacity, leading to the conclusion that pulmonary function may be influenced by the initial severity of the SARS‐CoV‐2 infection. Notably, this study included patients who experienced severe COVID‐19 (approximately 30% of the sample), shedding light on the potential consequences of this condition.

Öztürk et al. administered LFT to 50 children aged 5–18 years old, 3 months after the SARS‐CoV‐2 infection [11]. The authors compared patients with and without persistent symptoms, showing that those with persistent symptoms had significantly lower Forced Expiratory Volume in 1 s on forced vital capacity (FEV1/FVC) and higher lung clearance index (LCI), suggesting that they had increased occurrence of a lung obstructive pattern and ventilation inhomogeneity. This study also highlighted a significant reduction in diffusion capacity measured by DLCO in patients with severe initial disease compared to milder SARS‐CoV‐2 infection. The authors reported their inability to contact patients who suffered from critical COVID‐19; consequently, these children could not be included in the study, and this could affect the validity and generalizability of the study.

Ipek et al. assessed the respiratory outcomes experienced by children with a history of hospitalization related to COVID‐19 [12]. Patients underwent spirometry at least 1 month after discharge. Thirty‐four children were studied and compared to a group of 34 healthy control subjects. The authors observed a mixed‐type respiratory pattern, defined by a reduced FEV1, FVC, and FEV1/FVC. The main limitation of this research is the lack of long‐term follow‐up. In fact, patients were assessed only 4 weeks after discharge, making it difficult to make inferences on the duration of lung function impairments. Patients with chronic diseases were excluded, as in most published studies.

Sansone et al. recruited 28 patients suffering from long COVID syndrome, which was defined as symptom persistence after 12 weeks or more, and 30 asymptomatic patients with previous COVID‐19, and compared their lung function after a median period of 4.5 months from the infection [13]. The authors found that lung function, investigated by spirometry, body plethysmography, DLCO, was normal and similar between the two groups. Children with long COVID had lower values of Fractional Exhaled Nitric Oxide (FeNO).

Fireman Klein et al. conducted a prospective observational study including children aged 5–18 years (n. 58) with previous COVID‐19 who underwent spirometry and 6MWT; they were assessed 1–6 months after the acute infection [14]. Most of the participants (85%) did not experience persistent respiratory symptoms, making this study one of the few concentrating on this group of patients. The authors reported normal results of spirometry and 6MWT, suggesting that respiratory function is usually not impaired by COVID‐19. It should be noted that, out of 1180 children with confirmed SARS‐CoV‐2 infection, only 58 were included in the study for various reasons (e.g., failed contact attempt, refusal to participate, or presence of exclusion criteria), resulting in a potential risk of selection bias.

Schoeffl et al. investigated the cardiopulmonary effects of COVID‐19 among children and adolescents; this cross‐sectional study included 20 children suffering from long COVID‐19 (defined as symptoms lasting more than 28 days) and 28 subjects who had proof of SARS‐CoV‐2 infection but did not meet post‐COVID‐19 criteria [15]. Post‐COVID‐19 was defined as the occurrence of signs and symptoms that persist, develop, or fluctuate after SARS‐CoV‐2 infection for at least 2 months and cannot be explained by an alternative diagnosis. The enrolled children underwent cardiopulmonary exercise testing on a treadmill. The authors found a reduced absolute peak oxygen consumption (V̇O2) in children with long COVID‐19 symptoms, even though this was not confirmed for the percentage of predicted values, suggesting that deconditioning influenced the onset of the experienced symptoms. However, neither pulmonary, nor cardiac parameters proved to be significantly different from a group with no symptoms. The main limitation of this study was the small sample size; furthermore, a single LFT was used to assess the study outcomes.

Onay et al. conducted a prospective, single‐center cohort study on 55 children with persistent respiratory symptoms after COVID‐19 [16]. Spirometry, 6MWT, lung volume tests, DLCO, and FeNO were performed at least 4 weeks after infection, with a median follow‐up of 85 days. A group of 55 healthy subjects was included as a control group. The authors showed that a restrictive pattern was significantly more likely in the long‐COVID group (defined as the persistence of respiratory symptoms for at least 4 weeks after the completion of the acute infection period). Clinical scores in the acute infection did not correlate with long‐term lung function, whereas the authors found an association between the severity of imaging patterns and the LFT impairments (reduced lung volume and expiratory flow). Furthermore, the authors reported associations between specific symptoms and respiratory outcomes: dyspnea was associated with lower DLCO values, while cough predicted lower exercise capacity. Compared to other studies, this one involved a wide range of LFT and considered a comparison between symptoms and imaging findings.

Wongwathanavikrom et al. presented the clinical characteristics of 116 participants affected by COVID‐19 pneumonia during the illness and their LFT at 3‐month follow‐up [17]. The authors reported three cases of restrictive patterns at spirometry, and one with a concomitant diffusion defect; notably, all of them had at least one comorbidity. Approximately 40% of participants suffered from long‐COVID (defined as signs and symptoms occurring during or after COVID‐19 lasting for more than 12 weeks and not explained by any alternative diagnosis). In a subgroup analysis comparing children with long COVID to those without, a significant difference in Forced Expiratory Flow between 25% and 75% of vital capacity (FEF_25‐75_) was reported, −0.41 ± 1.19 versus 0.89 ± 0.94, respectively, suggesting an impairment in expiratory flow. It should be noted that comorbidities such as asthma, congenital defects, and prematurity were not deemed exclusion criteria in this study, therefore the reported outcomes may have been influenced by this fact and eventually reflect a real‐life context.

### 3.2 Studies With Long Term Follow Up

Palacios et al. carried out pre‐ and postbronchodilator spirometry, plethysmography, and diffusion capacity on 82 subjects (median age 15.2 years) with persistent pulmonary symptoms after COVID‐19 [18]. The tests were performed at two different time points, 3.5 months and 6.5 months after SARS‐CoV‐2 infection. The authors reported a normal spirometry in 77% of patients, whereas approximately 15% (*n* = 14) had obstructive deficits, and five patients showed restrictive deficits. Other findings were no abnormalities in plethysmography and diffusion capacity, and a positive bronchodilator response in 31% of patients. Eighty‐five percent of treated individuals reported a clinical response to therapy at follow‐up, including improved pulmonary function testing. It should be highlighted that the recruited patients were predominantly caucasians, privately insured, competitive athletes, and this could represent a population bias.

In a multicentre prospective observational study by Doležalová et al., including 39 children aged 2–18 years (median age 13.5) with persistent respiratory symptoms lasting more than 12 weeks after COVID‐19, normal lung function parameters were recorded 6 months after the infection in most children [19]. However, transient obstructive or restrictive ventilatory patterns and a mild decline of diffusing lung capacity were found in 5 and 3 children, respectively. The strength of this study lies in the national, multicentre design: on the other hand, this characteristic leads to some limitations, such as the differences in the laboratory equipment and software used by the different centers.

Another study comparing children affected by COVID‐19 pneumonia and controls was conducted by Bogusławski et al. [20]. The authors collected data from 41 children aged 0–18 who presented with COVID‐19 pneumonia and were therefore hospitalized during the first year of the pandemic. The authors performed clinical assessment, lung ultrasound, and LFT 3 months after discharge. Persistent symptoms were reported by 17% of patients, abnormal lung ultrasound was found in 63%, whereas impaired LFT were observed in 16% of children, mainly those with a history of severe illness. However, the authors did not report any significant difference in LFT data between the study group and the control group (including spirometry, body plethysmography, DLCO, and impulse oscillometry—IOS). Children with abnormal LFT at the first follow‐up visit underwent another assessment after 3 months. The authors reported no differences in LFT data between the 3‐month and 6‐month follow‐up visits.

Di Chiara et al. examined 61 children (6–18 years old) with a previous diagnosis of asymptomatic or mild COVID‐19 and reported normal spirometry outcomes 6–14 months after recovery [21]. Four patients underwent a bronchodilator reversibility test, with negative results. No correlation was found between LFT data and the number of months since infection, and no differences were found comparing children from 6 to 10 years of age with children aged > 10 years old (*n* = 28, 46%). Limitations of this study are the following: only spirometry was performed on patients, whereas pulmonary gas exchange capacity was not considered; moreover, results are only applicable to patients with mild and asymptomatic COVID‐19. One strength, on the other side, is that the authors performed a subanalysis based on different age groups, instead of generalizing the results.

Bode et al. recruited 25 children aged 8–14 years old (plus another 129 patients aged > 14 years) with functional respiratory disorders persisting approximately 1 year after mild SARS‐CoV‐2 infection [22]. The authors tested the subjects through spirometry and body plethysmography, performed either while resting or after standardized treadmill exercise, reporting normal findings. Notably, asthmatic patients were not excluded from this retrospective, single‐center study.

Iovine et al. performed spirometry in 433 of 589 patients aged 0–18 years (mean age 9.6 years) with previously confirmed SARS‐CoV‐2 infection, regardless of symptoms [23]. The mean duration of follow‐up was approximately 6 months. This study found no association between SARS‐CoV‐2 infection and long‐term impaired lung function: in fact, no patient had reduced forced vital capacity, and only 3.2% of subjects had a FEV1 < 80%. Comparing symptomatic versus asymptomatic infections, no differences were found in any respiratory outcome, suggesting that symptomatic COVID‐19 might not represent a significant risk factor for subsequent respiratory function impairment. This study mostly investigated children with previous mild or asymptomatic infections, excluding severe infections; thus, the results cannot be applied to the whole, heterogeneous population of children with a history of COVID‐19. Furthermore, no tests other than spirometry were performed, leading to a limited number of respiratory outcomes, and there was no comparison with healthy controls. On the other hand, its strengths are the large sample size and the long duration of follow up.

Shmueli et al. investigated lung function in 184 children aged ≤ 18 years old (mean age 12.4) with post‐COVID‐19 condition (PCC) [24]. The authors defined PCC as the presence of symptoms lasting more than 12 weeks after COVID‐19; different LFT (spirometry, exercise challenge test, bronchodilator response, lung plethysmography, FeNO, and MBNW) were performed. All participants were studied at baseline (25.1 weeks after COVID‐19), whereas 33 were followed up and underwent LFTs 13.6 weeks later. The authors described a mild obstructive pattern (characterized by reversibility post bronchodilators) in 11% of subjects. Lung volumes and diffusion were normal in all but one patient (1/134, 0.7%). Exhaled nitric oxide levels were elevated in 32/144 (22%). Improvement was demonstrated during follow‐up: all of the children had normal or near‐normal LFT on follow‐up testing, including those who showed an obstructive pattern at baseline. No comparison was made with healthy controls.

Sharanya et al. assessed lung function post‐SARS‐CoV‐2 infection in 40 children aged 7–18 years at 6‐month follow‐up, finding altered spirometry in one‐third of the sample [25]. In this descriptive study conducted in India, most of the alterations observed were restrictive, with reduced FVC and normal FEV1/FVC, especially in underweight patients. A limitation of this study consists of the use of Caucasian prediction equations to obtain the predicted normal spirometry values, in a population of children of Indian ancestry. Furthermore, no information about previous lung complications was available, making it questionable to consider the findings as surely related to COVID‐19.

Hevroni et al. recently published an observational case‐control study, comparing LFT results between infants experiencing persisting respiratory symptoms after COVID‐19 and a group of children assessed before 2019 (historical controls) [26]. The median age of cases and controls at the time of evaluation was 10.5 months and 9.4 months, respectively. LFT evaluation was performed 5.5 months after COVID‐19, by means of tidal breathing analysis and plethysmography. No significant differences were found between the two groups. Although this is one of the few studies conducted among infants, there were only 16 cases, whereas there were 475 controls. Thus, the generalizability of the study needs further confirmation.

The findings of the included studies are provided in Table 1.

**Table 1:** Details of the studies included in this systematized review.

	Number of patients (age)	COVID‐19 severity	LFT	Timing of assessment after COVID‐19	Main results
Ashkenazi et al. [7]	90 (mean 12 years)	Mild (moderate/severe in 8.9%)	Spirometry, plethysmography, DLCO	3.7 months	Abnormal spirometry (mild obstruction) in 45%, air trapping at plethysmography in 27%, abnormal DLCO in 2%
Leftin Dobkin et al. [8]	29 (mean 13.1 years)	Mild	Spirometry, plethysmography, DLCO, 6MWT	3.2 months	Spirometry: obstruction in 10.7%; plethysmography obstruction in 28.6%; 6MWT revealed exercise intolerance tachycardia in two‐thirds
Bottino et al. [9]	16 (median 7.5 years)	Mild/asymptomatic	Spirometry, DLCO, Rint	2 months	Normal LFT findings
Knoke et al. [10]	73 (5–18 years) + 45 controls	Mostly asymptomatic	Spirometry, plethysmography, DLCO, MBNW	2.6 months	Impaired LFT: spirometry 0%, MBNW 4.4%, plethysmography 7%, DLCO 5.3%
Öztürk et al. [11]	50 (median age 15 years)	Severe in 20% of the cohort	Spirometry, DLCO, MBNW	3 months	LFT impairment in 14%. Spirometry: obstruction in 6%; DLCO: impairment in 10% (30% of those with severe COVID‐19).
Ipek et al. [12]	34 (mean age 12.7 years) + 33 controls	Not specified	Spirometry	1.6 months	Mixed obstructive/restrictive impairment significantly more frequent in cases vs. controls
Sansone et al. [13]	28 with long COVID‐19 + 30 asymptomatic controls with previous COVID‐19	Not specified	Spirometry, plethysmography, DLCO, FeNO	4.5 months (median)	Reduced FEV1 in ≈10%; lower FeNO values in children with LCS than those without LCS
Fireman Klein et al. [14]	58 (5–18 years)	Asymptomatic 24%, mild 64%, moderate 12%	Spirometry, 6MWT	1–6 months	No significant LFT alterations
Schoeffl et al. [15]	20 with LCS (median age 13.5 years) + 28 controls	Not specified	Gas exchange, cardiopulmonary exercise test	Not specified	Almost all participants achieved maximal exercise. Reduced absolute peak oxygen consumption (V̇O2) in children with LCS vs controls. No significant differences between groups in the pulmonary variables
Onay et al. [16]	55 with LCS + 55 controls	Mild 62%; moderate pneumonia 33%, severe pneumonia 5%	Spirometry, plethysmography, DLCO, 6MWT, FeNO	2.8 months	Spirometry: 27% of cases vs 9% of controls had restrictive pattern; 10% vs. 6% had obstructive pattern. DLCO: low values in 16% vs. 0. 6MWT: lower distance in cases vs. controls. FeNO: non‐significant differences
Wongwathanavikrom et al. [17]	116 (median age 7 years) with COVID‐19 pneumonia	Severe pneumonia: 6%	Spirometry, DLCO, 6MWT	3 months	Restrictive ventilatory defects in 3 cases (1 with a concomitant diffusion defect. 6MWT: exercise intolerance in 43%)
*Studies with long‐term FUP (≥ 5 months)*					
Palacios et al. [18]	82 (mean 15.2 years)	Mild (8.5% hospitalized)	Spirometry, plethysmography, DLCO, 6MWT	3.5 and 6.7 months	Spirometry: 15% obstructive, 8% restrictive; no alterations in plethysmography/DLCO; most 6MWT with high symptom scores
Doležalová et al. [19]	39 (2–18, median 13.5)	Mild (but persisting dyspnea, cough, or chest pain after 3 months)	Spirometry, DLCO, 6MWT	6 months	Abnormal spirometry in 5 children (3 obstructive, 2 restrictive); abnormal 6MWT (low oxygen saturation) in 3 children; abnormal DLCO in 3 children (reduced diffusion)
Bogusławski et al. [20]	41 (0–18, median 3.7 years)	Moderate‐severe pneumonia	Spirometry, body plethysmography, DLCO, IOS	3 months and 6 months (in case of LFT impairment)	Impaired LFT in 5 children (3 restrictive and 2 obstructive)
Di Chiara et al. [21]	61 (10.9 ± 2.9 years)	Mild symptoms in 61%, asymptomatic in 39%	Spirometry	10 ± 4 months	Normal LFT data
Bode et al. [22]	53 (median age 9.4 years)	Not specified	Spirometry	12 months	Normal LFT data
Iovine et al. [23]	433 (0–18 years)	Asymptomatic/mild	Spirometry	5.7 months	Obstructive pattern in 3%
Shmueli et al. [24]	184 (mean age 12.4 years)	Asymptomatic 4.9%; mild 87.5%; moderate 4.9%; severe 2.7%	Spirometry, plethysmography, DLCO, MBNW, FeNO	After 6 months and after another 3 months if impaired LFT	At baseline LFT, 21.4% had mild obstructive pattern. Subsequent normalization was found afterwards
Sharanya et al. [25]	40 (7–18 years)	Moderate/severe in 37.5%	Spirometry	6 months	LFT impairment in ≈30% of cases (restrictive alterations)
Hevroni et al. [26]	16 (< 1 year)	Asymptomatic/mild (1 had ARDS)	Tidal breathing analysis, plethysmography, rapid thoraco‐abdominal compression techniques	5.5 months	LFT data were similar to a historical cohort from the pre‐COVID era

Abbreviations: 6MWT, 6‐minute walking test; DLCO, diffusing lung capacity for carbon monoxide; FeNO, fractional exhaled nitric oxide; IOS, impulse oscillometry; LCS, long‐COVID syndrome; MBNW, multiple breath nitrogen washout; Rint, interrupter resistance.

## 4 Discussion

In this review, we aimed to summarize data on respiratory function among infants and children with previous COVID‐19. There was a wide variability in the reported studies, particularly regarding the initial COVID‐19 severity, the type and timing of LFT, the age range of patients, and the duration of follow‐up. However, most studies assessed children with asymptomatic or mild COVID‐19 and included spirometric data. Eleven studies considered a short‐term follow‐up (< 5 months after COVID‐19), nine reported on a longer follow‐up.

Some studies reported no clear association between lung function impairment and a previous history of COVID‐19. Other authors reported mild, transient consequences, in particular obstructive or restrictive patterns demonstrated by spirometry. Three studies found post‐bronchodilator reversibility in patients with evidence of a mild obstructive pattern, suggesting that the administration of bronchodilators and inhaled corticosteroids could be useful in selected cases [8, 18, 24]. Potential risk factors for airway obstruction were older age and chest X‐ray findings of prominent bronchovascular markings and hyperinflation. Considering that COVID‐19 in children is usually mild and self‐remitting, little information was found with regards to the effects of severe SARS‐CoV‐2 infection on long‐term lung function in children. A small number of studies focused on the exiguous but relevant fraction of patients who suffered from severe COVID‐19, and observed different patterns: on one hand, Boguslawski et al. showed impaired LFT in 12% of children, similarly to healthy children [20]; on the other hand, other authors showed reduced forced vital capacity at follow up [10], restrictive patterns [16, 17, 25], and a significant decrease in DLCO in patients with severe disease compared to those with mild infection [11]. In one of the largest studies, one out of five children had a transient obstructive pattern, but there was no significant association between the severity of COVID‐19 and LFT data, except for a higher LCI in children with severe SARS‐CoV‐2 infection [24]. It could be speculated that these findings may be related to a variety of mechanisms, including impairments at the alveolar‐capillary membrane, airway, and respiratory muscular level, and possibly genetic factors in some children with severe COVID‐19.

A large portion of the studies evaluated children with prolonged symptoms (the so‐called long COVID or post‐COVID‐19 condition); little evidence has been published on respiratory sequelae of COVID‐19 in children who did not experience persisting symptoms; still, the results are reassuring in this specific population, as stated by some authors [11, 14].

Most of the papers included a small sample size of patients. Furthermore, the age range was usually wide, thus limiting the assessment of outcomes in specific age groups. Nevertheless, in one study, infants aged less than 12 months were evaluated [26], whereas in another study, two age groups were compared [21]. Overall, studies in early infancy showed normal lung function after COVID‐19.

There are some limitations regarding the present study: in particular, the heterogeneity of studies was high, particularly regarding the COVID‐19 severity, the included age groups, the LFT details, and the duration of follow‐up. Furthermore, the potential role of the different SARS‐CoV‐2 variants has not been evaluated, even though each study recruited patients in specific periods, each characterized by the epidemiological prevalence of a particular variant. Patients with comorbidities such as asthma or cystic fibrosis were usually excluded from the articles included in our review, and their respiratory trajectories are extensively treated in some specific studies.

Some factors could be advocated for future research: the design of longitudinal multicenter studies, the adoption of standardized follow‐up protocols, and the implementation of harmonized lung function assessment methodologies. Furthermore, the use of advanced computational techniques such as topological data analysis and machine learning could be tested to enhance the accuracy of COVID‐19 diagnosis [27, 28].

## 5 Conclusions

Even though persistent symptoms and LFT impairments are a possible consequence of COVID‐19 in otherwise healthy patients, this review showed that many children had normal long‐term lung function outcomes, except for a subgroup of those with severe COVID‐19. Further studies with a more homogeneous design are needed to confirm these findings.

## Author Contributions

**Benedetta De Santis:** writing – original draft, methodology, validation. **Francesca Baldo:** writing – original draft, methodology, data curation. **Giovanni Vento:** writing – review and editing, visualization, supervision. **Stefano Nobile:** writing – original draft, conceptualization, methodology, writing – review and editing.

## Funding

The authors have nothing to report.

## Conflicts of Interest

The authors declare no conflicts of interest.

## Transparency Statement

The corresponding author, Stefano Nobile, affirms that this manuscript is an honest, accurate, and transparent account of the study being reported; that no important aspects of the study have been omitted; and that any discrepancies from the study as planned (and, if relevant, registered) have been explained.

## Data Availability

Data sharing is not applicable to this article as no datasets were generated or analyzed during the current study.
